# Double Burden of Malnutrition among Bangladeshi Women: A Literature Review

**DOI:** 10.7759/cureus.1986

**Published:** 2017-12-26

**Authors:** Mehedi Hasan, Ipsita Sutradhar, ASM Shahabuddin, Malabika Sarker

**Affiliations:** 1 James P Grant School of Public Health, BRAC University

**Keywords:** double burden of malnutrition, women, and bangladesh

## Abstract

A narrative review was carried out of existing literature comprising nationally representative data. We searched PubMed, Google Scholar, and Banglajol databases. Quantitative studies reporting the prevalence and risk factors of the double burden of malnutrition (DBM) among Bangladeshi women based on nationally representative data were considered for this review. We included studies published between 1^st^ May 2007 and 30^th^ April 2017 in English language. Two researchers individually searched and screened all the relevant articles and separately extracted data using a data extraction table created in Microsoft Excel. Another researcher cross-checked the whole process to maintain consistency. Any sort of disagreement was resolved by group consensus. Thematic analysis was performed for data analysis. According to the included studies, the prevalence of underweight and stunting dramatically reduced among Bangladeshi women in last 10 years, though, nearly one-fourth of women are underweight and one-fifth of women are stunted in Bangladesh. Additionally, nearly half of the country’s women are suffering from different micronutrient deficiencies. This immense burden of undernutrition is accompanied by the presence of overweight or obesity among nearly half of the adult women. Women’s age, area of residence, education and wealth index have a significant influence on determining their nutritional status. DBM is an inevitable reality among Bangladesh women. The adverse health consequences of women’s undernutrition and overnutrition have been well documented. As women’s nutritional status is a multifaceted issue, effective implementation of very specific and focused public health interventions with inclusive multi-sectoral and multi-stakeholder approaches are indispensable to combat this problem.

## Introduction and background

Double burden of malnutrition (DBM) is an emerging public health concern nowadays which takes place as an inevitable consequence of nutritional transition [1]. Generally, a country is considered to endure DBM when undernutrition and overnutrition co-exist there within the same group of population [2]. DBM is indicated as the feature of low- and middle-income countries (LMICs) across the globe and Bangladesh is not an exception in this regard [3]. In Bangladesh, even though undernutrition constitutes enormous burden especially among women and children, the prevalence of overweight and obesity amid them is not negligible too [4].

Both nutritional deficiency and overnutrition are evident to have an adverse impact on human health [5,6]. People having nutritional deficiency are less immune and thus more prone to suffer from different infectious diseases [5]. On the contrary, overweight and obese persons are at higher risk to develop non-communicable diseases (NCDs) such as diabetes, hypertension, coronary heart disease and cancers [6]. Effects of malnutrition are further devastating for women because it not only affects their own health but also the health of their offspring. Malnourished women are also more susceptible to experience complications related to pregnancy and childbirth [7,8]. Hence, it is imperative to ensure proper nutrition for every woman in order to nurture a healthy nation.

For a country like Bangladesh, which is already troubled with an array of social, political as well as health issues, it is difficult to address double burden of malnutrition especially among women if its current trend and distribution are not properly known. Therefore, this study aimed to document the prevalence and risk factors of malnutrition encompassing both undernutrition (underweight, stunting, and micronutrient deficiency) and overnutrition (overweight and obesity) prevailing among Bangladeshi women. This study expectedly will offer a strong insight to the pertinent stakeholders of this country regarding the trend and magnitude of different forms of malnutrition existing among Bangladeshi women. This study also intended to offer some recommendations for policy and practice to address DBM in Bangladesh, which will provide some evidence to the stakeholders for planning and implementing target specific and focused public health interventions to overcome this burden.

## Review

Materials and methods

Search Strategy

We searched PubMed, Google Scholar, and Banglajol databases to identify relevant publications on DBM in Bangladesh. The team searched relevant articles using following keywords: "double burden of malnutrition", “DBM”, "dual burden of malnutrition", "nutritional transition", "underweight", "undernutrition", "overweight", "obesity", "micronutrient deficiency" "prevalence", "burden", "risk factors", "Bangladesh". In addition, we manually searched the bibliography of the finally selected articles or reports (snowballing) to identify more potential articles and reports related to DBM in Bangladesh.

Inclusion and Exclusion Criteria

All the articles were screened based on predefined inclusion and exclusion criteria. Inclusion criteria for this review were: (a) study or report published between 1^st^ May 2007 and 30^th^ April 2017 to get the most recent view on the topic, (b) published in English language, (c) quantitative study, (d) reported prevalence or burden and/or risk factors of and/or trend of double burden of malnutrition/undernutrition/underweight/overweight and obesity/micronutrient deficiency among Bangladeshi women, and (e) reported based on nationally representative data obtained through nationwide survey/surveillance. Exclusion criteria for this review were: (a) qualitative and review paper, (b) merely reported prevalence or burden and/or risk factors of underweight, and (c) absence of nationally representative data. Two researchers (first^ ^and second^ ^author) individually searched and screened the attained literature and another researcher (third^ ^author) cross-checked the whole process to maintain consistency. Any sort of disagreement was resolved by group consensus (All the authors).

Data Extraction and Analysis

Data extraction form was developed in Microsoft Excel format which includes: (a) title, (b) journal name (when applicable), (c) year of publication, (d) type of study, (e) data source and year of data collection, (f) study setting, (g) study population, (h) sample size, (i) prevalence of underweight/stunting/micronutrient deficiency/overweight and obesity, and (j) risk factors of underweight/stunting/micronutrient deficiency/overweight and obesity. Two researchers separately extracted data using the data extraction file which was again checked by each other and then by another researcher. We finally analyze the data under two broad themes: prevalence of malnutrition (undernutrition and overnutrition) and risk factors of malnutrition (undernutrition and overnutrition).

Quality Appraisal

We used a quality assessment checklist comprising five criteria to evaluate the quality of attained studies. The checklist was comprised of following points:

§  Clearly defined study objective.

§  Clearly mentioned study design.

§  Used random sampling technique.

§  Comprising adequate sample size.

§  Clearly described statistical analysis.

These criteria were developed based on the standards described in previous studies for quality assessment of published research [9-11]. Studies which fulfilled all five criteria were considered as high-quality studies. Studies that met three or four criteria were considered as moderate quality studies. The study which fails to fulfill less than three criteria was considered as a low-quality study.

Results

Search Result

**Figure 1:**
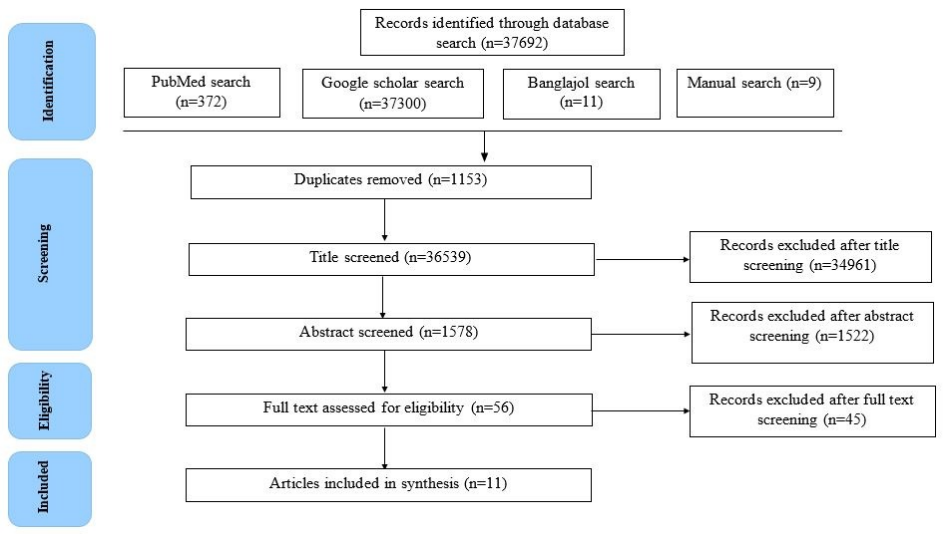
PRISMA diagram showing study selection process. PRISMA: Preferred reporting items for systematic reviews and meta-analyses.

Initially, 37,692 studies were identified from three electronic databases. After removal of duplications, the title was screened for 36,539 studies and abstract/executive summary was screened for 1578 studies. Finally, 56 studies remained for full-text assessment. However, among these, 11 relevant studies were finally eligible and included for the synthesis. The overall study selection process is displayed using Preferred Reporting Items for Systematic Reviews and Meta-Analyses (PRISMA) flow diagram (Figure 1).

Description of the Studies Included

Among the selected studies, six articles were published in peer-reviewed journals [12-17], however, rests were the reports of different nationally representative survey/surveillance conducted in Bangladesh [18-22]. The majority of the studies (nine studies) were published in 2011 and onwards, of which three were published within last two years (2015 and 2016) [14,20,21]. All the journal articles were based on secondary analysis of Bangladesh Demographic and Health Survey (BDHS) data between the year 1996 and 2011 [12-16] except one article that used primary data of a nationally representative household survey in rural Bangladesh [17]. Each of the reports included in this review was prepared based on primary data found through a nationally representative survey (National Micronutrients Status Survey) [22] or surveillance like Food Security and Nutrition Surveillance Project (FSNSP) and Food Security and Nutrition Surveillance-National Nutrition services (FSNS-NNS) project [18-21]. All the articles and reports included study population from both urban and rural settings except two articles, one study was conducted among rural women [17], whereas other study considered urban women during secondary analysis [15]. Study population varied from study to study. Ever-married, non-pregnant women (10–49 years) [15,16]; reproductive age women (15-49 years) [12]; ever-married women (15-49 years) [14]; non-pregnant non-lactating (NPNL) reproductive age women (15–49 years) [22]; adult women (19-49 years) and adolescent girls (10-18 years) [18-21]; and mothers of children aged 6–59 months were the study population of selected studies [17]. For the published articles, sample size ranged between 3634 and 1,68,317, however, for survey reports, the sample size was between 1050 and 22,713.

Quality of the Included Studies

All the six articles and five reports included in this review were of high or moderate quality considering the presence of clear objective, clearly mentioned study design, random sampling technique, adequate sample size comprising nationally representative data, and clearly described statistical analysis to identify prevalence/trend and risk factors (pooled meta-analysis, trend analysis, and binomial regression, multinomial logistic regression) of DBM among Bangladeshi women.

Prevalence of Underweight

In the year 1996-1997, more than half of reproductive age women (15-49 years) were underweight (Body mass index or BMI < 18.5 kg/m^2^) in Bangladesh [12]. This prevalence dramatically reduced, almost by 50%, in the following 10-12 years and in 2011, nearly one-fourth (24.0%) of them were reported as being underweight [13]. However, the slightly higher prevalence of underweight was reported in FSNSP report may be due to the inclusion of adolescent girls (10-18 years) along with adult women (19-49 years) in their study [18]. According to this report, in 2011, 32.5% women (adult women-22.0%, adolescent girls-43.0%) were underweight [18]. This prevalence continued to reduce among adult women throughout the subsequent years, however, remained almost unchanged for adolescent girls. In 2013, 17.0% adult women and 42.0% adolescent girls were reported to be underweight [19]. This prevalence reduced and became 16.0% and 36.0% respectively for adult women and adolescent girls in the year 2015 [20].

Factors Associated with Underweight

Women’s nutritional status is an outcome of various individual, familial and socio-cultural factors [14]. A number of studies found that level of education and wealth index play a vital role in shaping women’s nutritional status [14]. Women having a lower level of education and from lower wealth index were more likely to be underweight [15,17]. Age was also found to be correlated with their body weight in some studies. In Bangladesh, younger women (<30 years) were at higher risk to be underweight than their elderly counterparts (>30 years) [14,16,17]. Area of residence and marital status, additionally, were found to influence the nutritional status of women [14,17]. Rural women were more likely to be underweight than the women living in urban areas [14,17]. Some studies also found that prevalence of underweight was more frequent among the widow, divorced and separated women compared to currently married women [17].

Prevalence of Stunting

Consistent with the prevalence of underweight, prevalence of stunting was also reduced among Bangladeshi women throughout the last decade. According to FSNSP report, 12.0% adult women (19-49 years) and 31% adolescent girls (10-18 years) had the inadequate height for their age in 2011 [18]. This prevalence remained same for adult women in next few years, but gradually reduced for adolescent girls. In 2015, 25.0% adolescent girls were reported as being stunted [20]. Overall, these results indicate that one-fourth of adolescent girls and more than one-tenth of adult women were stunted in Bangladesh.

Factors Associated with Stunting

In addition to genetic, environmental and nutritional factors, women’s socio-economic background plays a vital role in determining their height [19]. Studies found that women’s level of education and wealth index influence their height [19]. Uneducated and poorer women were more likely to be short in height than educated and rich women [21]. This prevalence was almost similar between women of urban and rural settings, though, some regional variation was observed. Among the adult women (19-49 years), the prevalence of stunting was maximum in Dhaka division (15%) and lowest in Sylhet (11%) and Rajshahi division (11%) [20]. Conversely, the prevalence of stunting among adolescent girls (10-18 years) was highest in Sylhet division (32%) and lowest in Khulna division (10%). The prevalence of stunting was found greater among adolescent girls who were involved with income-generating work (41%) than those who did not have any income (24%) [20].

Prevalence of Micronutrient Deficiencies

The only micronutrient survey conducted in Bangladesh reported that 42.4% of all Bangladeshi women and 40.0% of NPNL reproductive age women (15-49 year) are suffering from anemia (Hb < 12 g/dL) [22]. This prevalence is much higher among pregnant women (49.6%). The same report also revealed that more than half (57.3%) of the NPNL reproductive age women (15-49 year) had zinc deficiency (S. zinc level <10.1 mmol/l), which is an essential micronutrient of the human body. According to National Micronutrients Status Survey 2011-12, iodine deficiency (UIC < 100.0 μg/l) was also prevalent among nearly half of these women (42.1%) [22]. At the national level, 5.4% NPNL reproductive age women (15-49 year) are reported to suffer from vitamin A deficiency (Serum retinol level < 0.7 mmol/l). Folate deficiency (S. Folate level < 6.8 nmol/l) and vitamin B12 deficiency (S. B12 level < 300.0 pg/ml) were also found among 9.1% and 22% of these women, respectively [22].

Factors Associated with Micronutrient Deficiencies

It was revealed from our review that iron deficiency anemia and iodine deficiency were more prevalent among rural women than women living in urban areas [22]. Anemia was also least prevalent among women with a higher level of education and belong to highest wealth quintile [14]. However, the prevalence of vitamin A deficiency and zinc deficiency was higher among women residing at slum area than rural and urban women. Interestingly, the prevalence of folate deficiency was found higher among urban women than their rural counterparts [22].

Prevalence of Overweight and Obesity

In the year 2000, only 5.0% reproductive age women (15-49 years) were overweight (BMI > 25 kg/m^2^) in Bangladesh [13]. This prevalence increased by more than three times in next decade and in 2011, 17.0% of them were reported as being overweight or obese [13,18]. The same study also stated that the prevalence of overweight (29.0%) was almost two times greater than the prevalence of underweight (14.0%) among urban women during the year of 2011-2013 [13]. This increasing trend of women’s BMI continued in succeeding years. According to FSNSP report, 20.0% and 24.0% adult women (19-49 years) were overweight/obese in the year 2012 and 2014, respectively [21]. Noticeably, in 2015, nearly half (41.0%) of this group of people was identified as having excessive BMI (>25 kg/m^2^) [20]. Similar trend of overweight and obesity was observed among adolescent girls (10-18 years); however, prevalence was considerably less among them than adult women (3.0% in 2011; 5.0% in 2013 and 7.0% in 2015) [18-20].

Factors Associated with Overweight and Obesity

A number of studies found that prevalence of overweight (BMI > 25 kg/m^2^) was higher among the urban women than those who live in rural areas [14,17]. Women having a higher level of education and belong to higher wealth index were at greater risk to be overweight and obese [15,17]. Women’s age was also found to be associated with their body weight in some studies. In Bangladesh, elderly women (>30 years) were more prone to be overweight and obese than younger women (<30 years) [14,16,17]. Interestingly, in one study, some other factors like residency in Dhaka division, highest neighborhood wealth, higher decision-making autonomy, belong to the Muslim family were identified as risk factors for women to be shifted from the state of underweight to overweight [17]. In urban settings, women from higher socio-economic status were more prone to be overweight and obese [15]. However, in rural settings, short stature, older and more educated women were more likely to be overweight and obese [16].

Discussion

To the best of our knowledge, this is the first review that documented the prevalence and associated factors of the double burden of malnutrition including underweight, stunting, micronutrient deficiency along with overweight and obesity among women of different age groups in Bangladesh. This review came up with several key issues which we discussed below.

The finding of this review indicates that, although the prevalence of undernutrition has been considerably reduced in last decade, still nearly one-fourth of the women in Bangladesh is underweight and a great portion of these women are short stature. Moreover, nearly half of the reproductive age women have different micronutrient deficiencies such as iron deficiency anemia, zinc deficiency, and iodine deficiency. This finding validates the results of a previous study identifying South-East Asian countries as the home of high and stagnant levels of undernutrition [23]. In line with the findings of a previous study conducted in India, our study also reveals that younger women, women from rural and urban slum area, women having a lower level of education and lower wealth index are more likely to suffer from different types of nutritional deficiencies [24]. This high prevalence of undernutrition among Bangladeshi women, especially amid poor, uneducated rural and slum women, can be explained by poor dietary intake (both quality and quantity) and discriminatory food allocation for females at household level [5]. Several socio-cultural and individual factors such as inadequate knowledge of diet and nutrition, lack of support from family members (husband, mother-in-law), low access to health care services and reluctance in seeking health care worsen this situation [5,25-27]. It is well established that undernourished individuals are more likely to suffer from a wide range of adverse health consequence compared to well-nourished people [5]. Underweight persons usually have less body immunity and, therefore, more vulnerable to different infectious diseases [5]. Stunting is positively associated with poor cognitive development, poorer school performance and poor adult income [28]. Iron deficiency anemia also reduces cognitive development and work performance of individuals [29]. Iodine deficiency impedes normal thyroid function of individuals [30] whereas zinc deficiency is associated with impaired physical growth and delayed sexual maturation [17,31-34]. Women’s undernutrition, in either form-underweight, short stature or micronutrient deficiency, is more crucial because it affects not only their own health but also the health of their offspring. Children of undernourished women are more vulnerable to impaired physical growth, poor cognitive maturity as well as development of chronic diseases (e.g., type 2 diabetes mellitus, hypertension, and cardiovascular disease) in their latter part of life [28,35,36]. Several studies have shown that stunted women more frequently give birth to low-birth-weight (LBW) baby and experience complication during pregnancy and delivery [28,37]. Micronutrient deficiency like chronic iron deficiency anemia has potential to increase the risk of hemorrhage and infection and thus to raise the chance of maternal mortality [38]. Pregnant women having iodine deficiency are at higher risk of development of fetal wastage and fetal cretinism [30]. Zinc deficiency is also associated with some pregnancy-related complications such as pre-eclampsia, eclampsia, premature rupture of membranes (PROM) and preterm delivery along with some complications among their babies like intrauterine growth retardation (IUGR) and congenital anomalies [39].

This review implies that the overweight and obesity prevalence among Bangladeshi women is being increased sharply and now one out of every four adult women are overweight or obese. This result supports the finding from previous studies where it was reported that the overweight and obesity prevalence has been increased drastically among the women of LMICs [40]. One possible reason for this trend might be within last few decades, per capita intake of rice, meat and fish has been increased remarkably among Bangladeshi people along with increased consumption of unhealthy fats, oils, and processed food though intake of fruits and vegetables is still suboptimal [41-43]. Inadequate physical activity, especially among women also might play role in this regard [44]. In the recent NCD risk factor survey of Bangladesh, it was reported that low physical activity was prevalent among 27.0% of the population where the majority were female (male: 10.5%, female: 41.3%) [44]. This review also shows that older women, urban women, women having a higher level of education and women from wealthier families are more likely to be overweight and obese. Prior studies conducted in LMICs also reported the similar finding that is rich and educated women from urban areas were more likely to be overweight and obese [24]. Sedentary and stressful lifestyle in combination with high-calorie intake by educated and wealthy women that developed as a result of rapid urbanization might explain this type of distribution of overweight [45]. Overweight and obesity have strong link with the development of different non-communicable diseases such as hypertension, diabetes mellitus, coronary heart disease, non-alcoholic fatty liver disease, gastroesophageal reflux disease [6,46]. In addition, studies found that overweight and obese women are at higher risk to experience pregnancy-related complications like preeclampsia, eclampsia and gestational diabetes mellitus (GDM). Overweight and obese women are also more likely to give birth through cesarean section [7]. This group of mothers is also at higher risk to deliver preterm baby and baby with congenital anomalies [8]. Similar finding was observed among Bangladeshi women too. A recent study conducted in Bangladesh found that overweight and obese women were at greater risk to develop pregnancy-related complications like gestational diabetes, hypertension and prolonged labor and more likely to deliver through caesarian section [47].

Recommendations for Policy and Practice

Our review suggests that it is a timely need for the policymakers and public health professionals to take necessary steps in order to prevent and control the double burden of malnutrition among Bangladeshi women. However, it is quite challenging to set intervention for a country where both undernutrition and overnutrition coexist, and because intervention to address one problem might exacerbate other. Therefore, target-specific intervention needs to be formulated and implemented. To address double burden of malnutrition, pertinent stakeholders need to establish multi-sectoral (within government organizations) and multi-stakeholder (public-private partnership and collaboration with development partners, civilians, and academic institutes) approach involving both government and private organizations [48]. To combat undernutrition, specific and focused public health programs are immediately required, e.g., raising awareness about detrimental health impact of undernutrition; increasing supply of healthy food items at lower cost; involving family members (specially husbands and mother-in-laws) while implementing nutritional programs among women; promoting water, sanitation and hygiene (WASH); ensure adequate nutrition-related logistic supply at health care facilities; and nutritional interventions in urban slums. It is also important to generate frontline community health workers whose main focus will be to improve the nutritional status of their communities. Strengthening nutrition-sensitive and nutrition-specific interventions, promoting dietary diversity, developing effective monitoring and evaluation system for nutrition-related sectors could be some other steps in this regard [48]. Further research is also warranted to identify implementation challenges that hinder the success of existing nutrition-related interventions in the context of Bangladesh. On the other hand, to address overweight and obesity, preventive programs through modifying a social and behavioral aspect of obesity can be adopted. For instance, educating and encouraging the affluent group of women to embrace a healthy lifestyle and generating awareness among them regarding the health impact of obesity using mass media; refashioning transport facility particularly in an urban area by making footpaths; providing a safe environment for women and adolescent girls for performing physical activities. Physicians and community health workers also can advise their patients, especially pregnant women so that women receive counseling about weight management before or during early pregnancy. The government should also take the initiative to restrict the production, purchase, and advertisements of junk food as well as to make fruits and vegetables accessible and affordable to the people of all socio-economic groups. Aggressive marketing strategy of multinational companies that promote various processed and junk food, moreover, needs to be confronted through multisectoral approach. Further research like a national survey to record overweight and obesity and to document dietary patterns of urban, rural and slum women will be helpful in this regard.

## Conclusions

DBM is an inevitable reality among women in Bangladesh. Currently, a vast majority of women are suffering from different nutritional deficiencies, which is burdened with the sharply rising trend of overweight and obesity. The detrimental consequences of women’s undernutrition and over-nutrition such as increased risk of different communicable and non-communicable diseases, reduced productivity, and adverse pregnancy outcomes have been well documented in the context of Bangladesh. Prevention and control of malnutrition among women have the potential to bring significant health and economic paybacks for the country. As women’s nutritional status is a multifaceted issue with a range of underlying biological and socio-cultural factors like age, area of residence, the level of education and wealth index, it is not possible to deal with DBM unless it becomes everybody’s business and responsibility. To serve this purpose, effective implementation of target specific and focused public health interventions with highly organized and inclusive multi-sectoral and multi-stakeholder approaches along with continuous monitoring and evaluation process are indispensable.

## References

[1] Daboné C, Delisle HF, Receveur O (2011). Poor nutritional status of schoolchildren in urban and peri-urban areas of Ouagadougou (Burkina Faso). Nutr J.

[2] Shrimpton R, Rokx C (2017). The double burden of malnutrition: a review of global evidence. https://openknowledge.worldbank.org/handle/10986/27417.

[3] Shukla HC, Gupta PC, Mehta HC (2002). Descriptive epidemiology of body mass index of an urban adult population in western India. J Epidemiol Community Health.

[4] Biswas T, Garnett SP, Pervin S (2017). The prevalence of underweight, overweight and obesity in Bangladeshi adults: data from a national survey. PLoS ONE.

[5] Kamal SM, Islam A (2010). Socio-economic correlates of malnutrition among married women in Bangladesh. Malays J Nutr.

[6] Burton BT, Foster WR, Hirsch J (1985). Health implications of obesity: an NIH consensus development conference. Int J Obes.

[7] Baeten JM, Bukusi EA, Lambe M (2001). Pregnancy complications and outcomes among overweight and obese nulliparous women. Am J Public Health.

[8] Marchi J, Berg M, Dencker A (2015). Risks associated with obesity in pregnancy, for the mother and baby: a systematic review of reviews. Obes Rev.

[9] Fowkes FG, Fulton PM (1991). Critical appraisal of published research: introductory guidelines. BMJ.

[10] Vandenbroucke JP, Von Elm E, Altman DG (2007). Strengthening the reporting of observational studies in Epidemiology (STROBE): explanation and elaboration. PLoS Med.

[11] Sanderson S, Tatt ID, Higgins J (2007). Tools for assessing quality and susceptibility to bias in observational studies in epidemiology: a systematic review and annotated bibliography. Int J Epidemiol.

[12] Balarajan Y, Villamor E (2009). Nationally representative surveys show recent increases in the prevalence of overweight and obesity among women of reproductive age in Bangladesh, Nepal, and India. J Nutr.

[13] Khan SH, Talukder SH (2013). Nutrition transition in Bangladesh: is the country ready for this double burden. Obes Rev.

[14] Kamal SM, Hassan CH, Alam GM (2015). Dual burden of underweight and overweight among women in Bangladesh: patterns, prevalence, and sociodemographic correlates. J Health Popul Nutr.

[15] Khan MMH, Krämer A (2009). Factors associated with being underweight, overweight and obese among ever-married non-pregnant urban women in Bangladesh. Singapore Med J.

[16] Oddo VM, Rah JH, Semba RD (2012). Predictors of maternal and child double burden of malnutrition in rural Indonesia and Bangladesh. Am J Clin Nutr.

[17] Corsi DJ, Kyu HH, Subramanian SV (2011). Socioeconomic and geographic patterning of under-and overnutrition among women in Bangladesh. J Nutr.

[18] James P Grant School of Public Health (JPGSPH), Helen Keller International (2017). State of food security and nutrition in Bangladesh 2011. http://sph.bracu.ac.bd/images/reports/FSNSP/State%20of%20Food%20Security%20&%20Nutrition%20in%20Bangladesh%202011.pdf.

[19] Helen Keller International, James P Grant School of Public Health (2017). State of food security and nutrition in Bangladesh 2013. http://sph.bracu.ac.bd/images/reports/FSNSP/state_of_food_security__nutrition_in_bangladesh_2013.pdf.

[20] James P Grant School of Public Health, National Nutrition Services (2017). State of food security and nutrition in Bangladesh 2015. http://sph.bracu.ac.bd/images/reports/FSNSP/state_of_food_security__nutrition_in_bangladesh_2015.pdf.

[21] Helen Keller International, James P. Grant School of Public Health (2017). State of food security and nutrition in Bangladesh 2014. http://sph.bracu.ac.bd/images/reports/FSNSP/state_of_food_security__nutrition_in_bangladesh_2014.pdf.

[22] (2017). National micronutrients status survey 2011-12. http://dspace.icddrb.org/jspui/handle/123456789/6450.

[23] Haddad L, Cameron L, Barnett I (2015). The double burden of malnutrition in SE Asia and the Pacific: priorities, policies and politics. Health Policy Plan.

[24] Subramanian SV, Smith GD (2006). Patterns, distribution, and determinants of under-and overnutrition: a population-based study of women in India. Am J Clin Nutr.

[25] Ahmed SM, Adams AM, Chowdhury M (2000). Gender, socioeconomic development and health-seeking behaviour in Bangladesh. Soc Sci Med.

[26] Nguyen PH, Sanghvi T, Kim SS (2017). Factors influencing maternal nutrition practices in a large scale maternal, newborn and child health program in Bangladesh. PLoS One.

[27] Paul BK, Rumsey DJ (2002). Utilization of health facilities and trained birth attendants for childbirth in rural Bangladesh: an empirical study. Soc Sci Med.

[28] Victora CG, Adair L, Fall C (2008). Maternal and child undernutrition: consequences for adult health and human capital. The Lancet.

[29] World Health Organization (2017). The world health report 2002 - reducing risks, promoting healthy life. http://www.who.int/whr/2002/en/.

[30] Dunn JT (1993). Iodine supplementation and the prevention of cretinism. Ann N Y Acad Sci.

[31] Rink L, Kirchner H (2000). Zinc-altered immune function and cytokine production. J Nutr.

[32] Blanchard RK, Cousins RJ (2000). Regulation of intestinal gene expression by dietary zinc: induction of uroguanylin mRNA by zinc deficiency. J Nutr.

[33] Black RE, Sazawal S (2001). Zinc and childhood infectious disease morbidity and mortality. Br J Nutr.

[34] Hambidge M (2000). Human zinc deficiency. J Nutr.

[35] Barker DJ (1992). Fetal growth and adult disease. BJOG Int J Obstet Gynaecol.

[36] Liu L, Johnson HL, Cousens S (2012). Global, regional, and national causes of child mortality: an updated systematic analysis for 2010 with time trends since 2000. The Lancet.

[37] Camilleri AP (1981). The obstetric significance of short stature. Eur J Obstet Gynecol Reprod Biol.

[38] Brabin BJ, Hakimi M, Pelletier D (2001). An analysis of anemia and pregnancy-related maternal mortality. J Nutr.

[39] Caulfield LE, Zavaleta N, Shankar AH (1998). Potential contribution of maternal zinc supplementation during pregnancy to maternal and child survival. Am J Clin Nutr.

[40] Popkin BM, Slining MM (2013). New dynamics in global obesity facing low-and middle-income countries. Obes Rev.

[41] Chen Y, Factor-Litvak P, Howe GR (2006). Nutritional influence on risk of high blood pressure in Bangladesh: a population-based cross-sectional study. Am J Clin Nutr.

[42] Jiang J, Liu M, Parvez F (2015). Association of major dietary patterns and blood pressure longitudinal change in Bangladesh. J Hypertens.

[43] World Health Organization, others others (2017). Diet, nutrition and the prevention of chronic diseases. Diet, nutrition and the prevention of chronic diseases: report of a joint WH.

[44] World Health Organization Bangladesh and Ministry of Health & Family Welfare, Bangladesh Bangladesh (2017). Non-communicable disease risk factor survey Bangladesh 2010. World Health Organization Bangladesh.

[45] Rahim MA, Hussain A, Khan AA (2007). Rising prevalence of type 2 diabetes in rural Bangladesh: a population based study. Diabetes Res Clin Pract.

[46] Hampel H, Abraham NS, El-Serag HB (2005). Meta-analysis: obesity and the risk for gastroesophageal reflux disease and its complications. Ann Intern Med.

[47] Khan MN, Rahman MM, Shariff AA (2017). Maternal undernutrition and excessive body weight and risk of birth and health outcomes. Arch Public Health.

[48] Hussain AZ, Talukder MQK, Ahmed T (2017). Nutrition background paper to inform the preparation of the 7th five year plan. http://www.plancomm.gov.bd/wp-content/uploads/2015/02/23_FINAL-Nutrition-Background-Paper-for-7th-Five-Year-Plan-_-23-Feb-2015.pdf.

